# Hofmeister Effects Shine in Nanoscience

**DOI:** 10.1002/advs.202302057

**Published:** 2023-05-21

**Authors:** Weichen Wei

**Affiliations:** ^1^ Department of Nanoengineering University of California San Diego La Jolla San Diego CA 92093 USA

**Keywords:** Hofmeister effect, hydrogel engineering, ion‐responsive nanosystems, nanotechnology, supramolecular chemistry

## Abstract

Hofmeister effects play a crucial role in nanoscience by affecting the physicochemical and biochemical processes. Thus far, numerous wonderful applications from various aspects of nanoscience have been developed based on the mechanism of Hofmeister effects, such as hydrogel/aerogel engineering, battery design, nanosynthesis, nanomotors, ion sensors, supramolecular chemistry, colloid and interface science, nanomedicine, and transport behaviors, etc. In this review, for the first time, the progress of applying Hofmeister effects is systematically introduced and summarized in nanoscience. It is aimed to provide a comprehensive guideline for future researchers to design more useful Hofmeister effects‐based nanosystems.

## Graphical abstract

Hofmeister effects play key and extensive roles in nanoscience. Numerous works are devoted to applying Hofmeister effects to the applications of nanoscience. In this article, for the first time, these applications are introduced and discussed including hydrogel/aerogel engineering, battery design, nanosynthesis, nanomotors, ion sensors, supramolecular chemistry, colloid and interface science, nanomedicine, nanotransport, etc with helpful outlook.

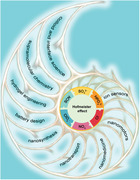

## 1 Introduction

**Figure 1:**
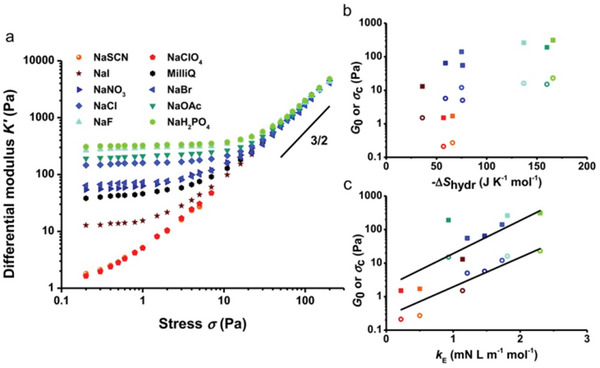
Nonlinear rheology of PIC gels with different sodium salts added. a) The differential modulus K′ as a function of the applied stress *σ* for gels with different sodium salts at a salt concentration of 0.5 m and T = 37 °C. Chaotropic anions decrease the linear modulus of the gel but increase the sensitivity toward applied stress, while kosmotropic anions increase the linear modulus and decrease the sensitivity toward stress. Note that all gels behave similarly at high stress. b) The plateau modulus G_0_ (squares) and the critical stress *σ*_c_ (open circles) for gels with different anions plotted versus the entropy of hydration of the anions. The colors correspond to the legend of the panel a). c) The plateau modulus G_0_ (squares) and the critical stress *σ*_c_ (open circles) for gels with different anions plotted versus the surface tension increments of the anions. The colors correspond to the legend of the panel a). Both G_0_ and *σ*_c_ show a remarkably good correlation for both chaotropic and kosmotropic anions. The solid lines represent fits with Equation (3). Reproduced with permission.^[^ ^20^ ^]^ Copyright 2015, Wiley‐VCH.

**Scheme 1:**
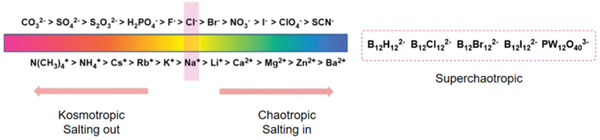
Modern version for Hofmeister series of anions and cations.

Ions can affect various aspects of water structure and dynamics.^[^ ^1^, ^2^ ^]^ In 1888, Franz Hofmeister established Hofmeister series (**Figure** **1**) with great scientific significance, that is, in salt solutions, different salt ions have different precipitation abilities to egg white proteins or other colloids.^[^ ^3^ ^]^ Hofmeister effects are mainly reflected by changing the physical and chemical properties of the solutes in solutions. Typically, the effect of anions is remarkably stronger than that of cations. The ions interacting strongly with water are called kosmotropes, and those interacting weakly with water are called chaotropes. Thus far, various theories have been proposed to elucidate Hofmeister effects. Though it is pretty challenging to completely solve the mystery of this complicated ion‐solvent‐colloidal macromolecule ternary system, researchers have made huge progress in molecular dynamics simulations and collecting experimental proofs to better understand the mechanism of Hofmeister effects (**Scheme** **1**).^[^ ^4^, ^5^, ^6^, ^7^, ^8^, ^9^, ^10^, ^11^, ^12^, ^13^, ^14^ ^]^

Hofmeister effects are typically assessed through experimental and theoretical methods. Dynamic light scattering (DLS), nuclear magnetic resonance (NMR) spectroscopy and dark field microscopy, atomic force microscopy (AFM), and nanopores are powerful experimental tools for studying the Hofmeister effects.^[^ ^9^, ^15^, ^16^, ^17^, ^18^, ^19^ ^]^ DLS can measure very quantitative information such as hydrodynamic diameters. NMR spectroscopy can study the binding affinity of ions in various systems. The combination of dark field microscopy and temperature gradient microfluidic device helps to study the mechanism of Hofmeister effects by exploring the phase transition process. However, these optical techniques are highly sensitive to concentrations, and it is quite challenging to overcome the interference caused by aggregation in high‐concentration systems, which is not conducive to exploring Hofmeister effects in polydisperse systems. Therefore, better choices for studying the Hofmeister effect at the single‐molecule level are AFM and nanopores. In addition, molecular dynamics (MD) simulation is an indispensable theoretical tool for studying Hofmeister effects. MD simulations have great advantages in studying solvation effects but cannot accurately describe quantum mechanical effects. Marrink developed and improved MARTINI force field, and in the newest version MARTINI‐3,^[^ ^14^ ^]^ the accurate simulation of Hofmeister effects is more systematic and the analysis of the abnormal Hofmeister effects is basically realized.

Hofmeister effects were originally related to the effects on bulk water, but this notion has been abandoned in the modern literature, especially for chaotropic anions, which also directly interact with dissolved matter. Over the years, with the development of nanoscience, Hofmeister effect has been widely used for various applications of nanoscience. In this review, for the first time, we systematically introduce and summarize the progress of applying Hofmeister effects to nanoscience and propose helpful and constructive comments and suggestions in the perspective part.

## 2 Hofmeister Effects in Hydrogel/Aerogel Engineering

### 2.1 Hofmeister Effects in Mechanical Properties of Hydrogels

Mechanical properties are the most important properties for hydrogel applications. Utilizing Hofmeister effects to regulate hydrogels does not require modification of raw materials or the addition of any chemical cross‐linking agents, providing a simple strategy to greatly enhance the mechanical properties of hydrogels. Kouwer and Rowan first systematically explored the impact of Hofmeister effects on the mechanical properties of hydrogels.^[^ ^20^ ^]^ The lower critical solution temperature of thermoresponsive hydrogels is changed to tailor the mechanical properties via Hofmeister effect. The addition of chaotropic ions will gain more sensitive and softer hydrogels, while the kosmotropic ions will induce the production of stiff hydrogels with less sensitivity (Figure 1). Such a strategy has technical merit in that the microstructure of gels will not change along with the mechanical properties.

**Figure 2:**
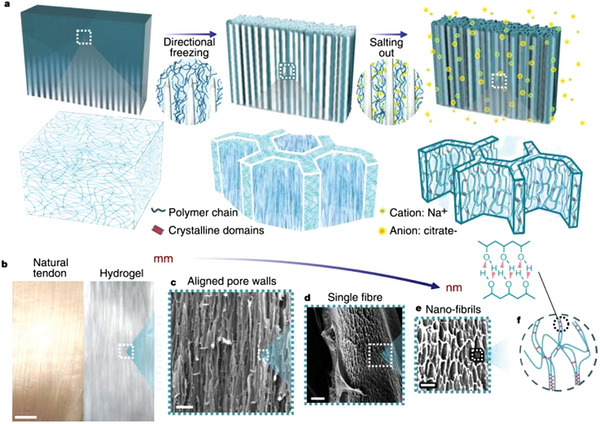
a) Freezing‐assisted salting‐out fabrication procedure of the HA‐PVA hydrogels. Structural formation and polymer chain concentration, assembly, and aggregation during the freezing‐assisted salting‐out fabrication process. b) Macroscopic view of real tendon and of the HA‐5PVA hydrogel. Scale bar, 5 mm. c–e) SEM images showing the microstructure c) and nanostructure d,e) of the HA‐5PVA hydrogel. Scale bars, 50 µm c); 1 µm d); 500 nm e). f) Molecular illustration of polymer chains aggregated into nanofibrils. Reproduced with permission.^[^ ^21^ ^]^ Copyright 2022, Springer Nature.

He proposed a new strategy to improve the performance of hydrogels—freeze‐casting assisted salting out, which can produce multi‐level anisotropic structures on different scales and significantly improve the mechanical properties of hydrogels (**Figure** **2**).^[^ ^21^ ^]^ Directional freeze‐casting enables hydrogels to have anisotropic structures on larger scales while increasing polymer concentrations. Modulus‐contrasting structures can be formed in the same polymer composition by changing the aggregation state of the polymer through salting out induced by Hofmeister effects. Here, these two strategies were pioneeringly combined. During the gel preparation, the PVA solution was directionally frozen first, then immersed in the kosmotropic salt solution for salting out. During the directional freezing process, a honeycomb micro‐network structure with well‐arranged pore walls is produced, and the polymer chains are condensed and packed more tightly. In the subsequent salting‐out process, the pre‐concentrated PVA chains would aggregate strongly, separate from the homogeneous phase, and form network nanofibers on the surface of the pore walls until the crystal structure was stable. With different kosmotropic ions, the microstructure of hydrogels obtained is also varied, so the mechanical properties of hydrogels can be tuned. Among them, sodium citrate has the best salting‐out capability, and the prepared PVA hydrogel has the highest elastic modulus, so it was selected as the kosmotropic ion used in this work. Control experiments show that the synergistic mechanical properties of the two strategies are better than the performance of only directional freezing or salting out. Hydrogels that were only directionally frozen did not form networked nanofibers, demonstrating a low degree of polymer chain aggregation. Salting out treatment only resulted in loose and randomly entangled fibers. These results indicate that the synergistic effect of directional freezing and salting out is the key to obtaining structure hydrogels with high strength, high toughness, high ductility, and hierarchical structure. Salting‐out treatment can increase the crystallinity, and the crystal domains are rigid and can act as cross‐links, which improves strength and toughness.

Subsequently, they applied a similar strategy to study the specific effect of Hofmeister ions on the gelation of PVA.^[^ ^22^ ^]^ The ability of different ions to improve the gelation and mechanical properties of PVA follows the following order: 1) anions: SO_4_^2−^> CO_3_^2‐^ > Ac^−^ > Cl^−^ > NO_3_^−^ > I^−^; 2) cations: K^+^ > Na^+^ ≈ Cs^+^ > Li^+^ ≈ Ca^2+^ ≈ Mg^2+^. By changing the type and concentration of salts, the mechanical properties of PVA hydrogels can be tuned in an extensive range, making it possible to meet the needs of various fields such as biomedical engineering, robotics, and wearable electronics.

**Figure 3:**
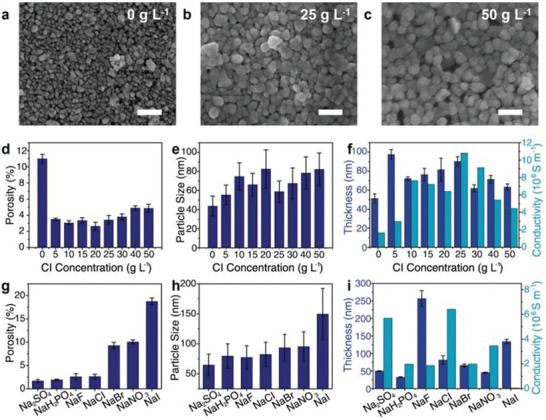
a–c) SEM image of printed Au using inks with NaCl concentrations of 0, 25, and 50 g L^−1^, respectively. d) Porosity as a function of Cl^−^ anion concentrations. e) Particle sizes as a function of Cl^−^ anion concentrations. f) Thickness and conductivity as a function of Cl^−^ anion concentrations. g) Porosity of samples with different anions. h) Particle sizes of samples with different anions. i) Thickness/conductivity of samples with different anions. Data represent mean ± standard deviation, *n* = 5, significance determined by one‐way ANOVA test. Scale bar: 200 nm for a–c). Reproduced with permission.^[^ ^23^ ^]^ Copyright 2022, Wiley‐VCH.

Furthermore, He achieved annealing‐free gold printing at room temperature by Hofmeister anion‐assisted photochemical deposition (**Figure** **3**).^[^ ^23^ ^]^ The type of Hofmeister anions in the precursor ink significantly affected tuning the printed pattern's morphology and conductivity. Au nanoparticle printing with high density and small size can be achieved in the presence of kosmotropes like SO_4_^2−^, while a loose network with larger Au nanoparticles can be obtained by adding chaotropes like I^−^. These kosmotropes can reduce the surface energy of Au nanoparticles to facilitate their growth and sintering. On the other hand, chaotropes tend to adsorb to the surface of Au nanoparticles, resulting in the repulsion of negatively charged Au nanoparticles. Despite a strong correlation between structure and anion species, the thickness and conductivity of printed Au are not closely related to Hofmeister series.

**Figure 4:**
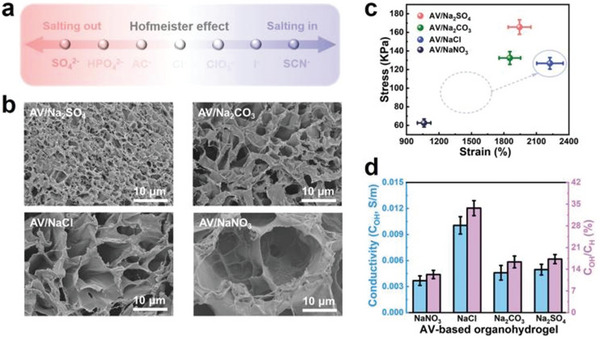
Impact of Hofmeister effects on the AV‐based organohydrogels: a) Schematic illustration of the simplified anionic Hofmeister series, b) SEM images, c) Stress and strain values, and d) Conductivity comparison of various sodium salt‐based AV organohydrogels. Reproduced with permission.^[^ ^25^ ^]^ Copyright 2022, Wiley‐VCH.

The problem of easy drying of hydrogels can be solved by using cryoprotectant replacement strategies. However, the induction of insulating solvents will lead to a decrease in the conductivity of organic hydrogels, which dramatically limits their electronic applications. Feng et al. used Hofmeister effects and electrostatic interactions to generate hydrogen/sodium bonds in hydrogels, combined with its double network to establish an effective charge channel that is not affected by solvent replacement, making it have better performance for electronic applications (**Figure** **4**).^[^ ^24^, ^25^ ^]^

Wang reported that Hofmeister effects of PVA was applied to improve the mechanical properties of hydrogels to prepare high‐performance hydrogel electrolytes and supercapacitors.^[^ ^26^, ^27^, ^28^ ^]^ They then developed the ionohydrogels based on Hofmeister effects as printable bioelectronic devices. [EMIM][EtSO_4_] acts as an ion source and bioprotectant, combining chaotropic cations, and kosmotropic anions to maintain a hydration layer and stabilize proteins. Therefore, it can ensure the mild gelation and the stability of the enzyme in situ immobilization, thus realizing the construction of an ionohydrogel bioelectronic platform by enzymatic polymerization. Furthermore, it can form ternary supramolecular interactions with water and polymer chains, thereby providing ionohydrogels with excellent mechanical properties and wide temperature windows, addressing the mismatch between tissues and artificial machines.

**Figure 5:**
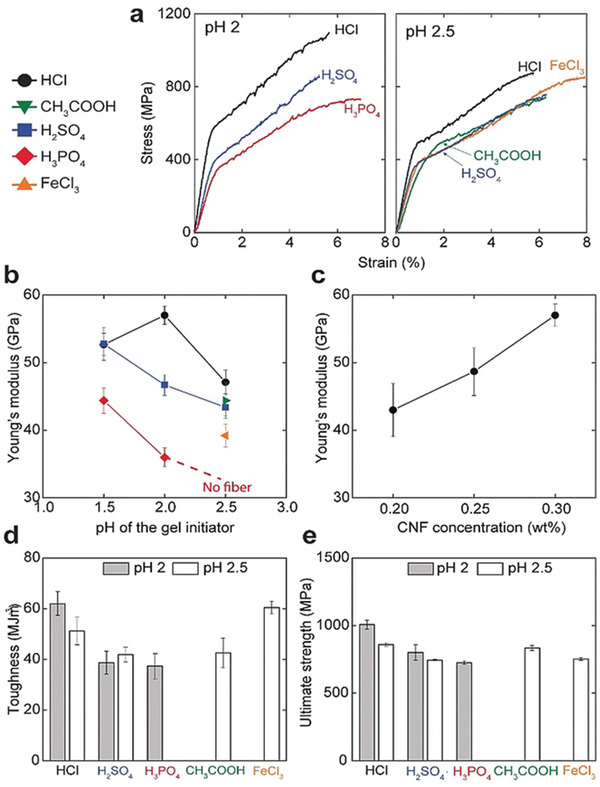
Tensile mechanical properties of CNF biofibers. a) Representative stress‐strain curves of fibers prepared at pH 2 and 2.5 of the gel initiators and 0.3 wt.% CNF concentration. Young's modulus as a function of b) pH of the gel initiator and, c) CNF concentration for HCl. d) Toughness and e) ultimate strength of fibers prepared with different gel initiators at pH 2 and 2.5. The different acids are referred to by the color and shape of the data points. Error bars are 90 % confidence intervals based on at least 10 different measurements for each type of sample. All the measurements are done at 50 % RH. Reproduced with permission.^[^ ^29^ ^]^ Copyright 2019, Wiley‐VCH.

Söderberg reported the microfluidics‐based assembly of strong, tough, and stiff nanofibrils induced by ion‐specific effects like hydration enthalpy and polarizability (**Figure** **5**).^[^ ^29^ ^]^ Three different cases, kosmotropic, chaotropic, and Lewis acids, were explored for the potential mechanism of gel network initiation. Since the cellulose surface is chaotropic, the chaotropic ions aggregate or adsorb on the cellulose surface, while the kosmotropic ions are dispersed in the bulk aqueous solution. Although Fe^3+^ is highly hydrated, due to the entropy increase effect of carboxyl groups in the system, it accumulates inside the hydrogel through electrostatic attraction and coordination bonds. Therefore, the relative acid concentration inside the gel and in the aqueous solution depends on the chaotropic/kosmotropic ions, which is similar to the fact that measuring the pH of a solution with a glass electrode is ion‐specific. Similarly, the effects of using acids with different anions as gel initiators are also different. Materials tests have shown cellulose nanofibrils fabricated with HCl are harder than those fabricated with H_2_SO_4_ and H_3_PO_4_. The chaotropic/kosmotropic nature of ions significantly affects the water absorption of the gel, thereby affecting the mechanical properties of cellulose nanofibrils. However, this is not the only determining factor, and further studies have shown that the polarizability of the ions, the ionization of the acid, the size of the ions, and the sensitivity of the gel surface (functional groups) to specific ions are all possible influencing factors.

Wang demonstrated the use of Hofmeister effects to improve the mechanical properties of gelatin hydrogels.^[^ ^30^ ^]^ Soaking gelatin hydrogels directly in (NH_4_)_2_SO_4_ or other kosmotropic salt solutions can endow gelatin hydrogels with super strong mechanical properties. Further study found that chain entanglement, hydrophobic interactions, and microphase separation regions in the hydrogel network may be caused by Hofmeister effects, resulting in a substantial increase in the mechanical strength of the hydrogel. However, when the hydrogel was treated with chaotropic salt solutions, the hydrogel softened and even dissolved. In addition to improving the mechanical properties, Hofmeister effects can simultaneously be used to improve the biocompatibility of hydrogels. Pan reported that Hofmeister effects of H_2_PO_4_^−^ were used to embed aldehyde groups in the center of entangled polymer chains, thereby improving the biocompatibility of gelatin/oxidized dextran hydrogels.^[^ ^31^ ^]^ In rat full‐thickness skin defect experiments, the hydrogel based on the H_2_PO_4_^−^ Hofmeister effects promoted wound healing and exhibited better histological morphology than ordinary hydrogels. Furthermore, such strength of Hofmeister effects has been employed to develop strong gelatin hydrogel actuators.

Liu exploited Hofmeister effects to strengthen the internal connections of collagen molten fibril networks.^[^ ^32^ ^]^ Kosmotropes (SO_4_^2−^, CO_3_^2−^) can strengthen the molten fibril network by enhancing the hydrophobic interactions between the films, while chaotropes weaken the hydrophobic interactions, leading to swelling or even dissolution of the material. The molten fibril network exhibits biomimetic stimulus‐hardening behavior after exposure to (NH_4_)_2_SO_4_ (strong kosmotropic salt) solution. In contrast, static fiber networks are less responsive because hierarchically organized fibers make full use of hydrophobic amino acid residues and are, therefore, less sensitive to lyophilic salts. In addition, the formation of covalent bonds restricts the free movement of collagen fibers is also a possible reason. Considering Hofmeister effects‐induced strengthening process of the molten fibril network is dynamically reversible, it has highly desirable biomedical applications, such as regulating blood vessels and blood flow as a degradable material.

More recently, the mechanical properties of PVA hydrogel have been applied to fabricate functional biomedical devices such as microneedles and pressure‐responsive iontronic sensors. Using a strategy combining template replication and 3D transfer printing, Zhao developed a novel biomimetic adaptive indwelling microneedle that encapsulates therapeutic exosomes and used it to promote diabetic wound healing.^[^ ^33^ ^]^ The microneedle consists of an adjustable PVA hydrogel tip encapsulating mesenchymal stem cell exosomes and a detachable 3 M medical tape support base. Due to Hofmeister effects, the mechanical strength of this PVA hydrogel is ion‐responsive, so the tip hardness of the microneedles can be upregulated by sulfate ions to ensure skin penetration. After the needle tip is detached from the substrate, nitrate ions can soften the needle tip hardness to adapt to the surrounding tissue and release exosomes.

**Figure 6:**
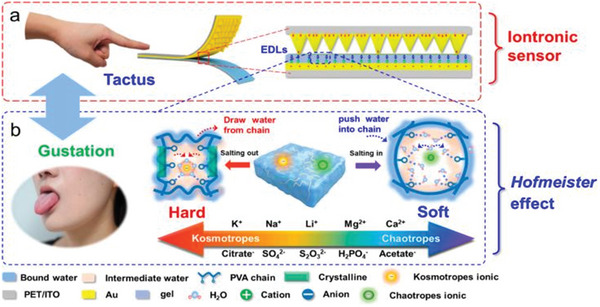
Schematic illustration of the Hofmeister gel iontronic sensor. a) The sandwiching capacitance with hierarchical pyramid electrodes and conductive hydrogel dielectric layer construct the gel iontronic sensor. b) The Hoffmeister effect in the hydrogel manages the gel elasticity modulus of the dielectric layer with the presence of various chemical cosolvents. Reproduced with permission.^[^ ^34^ ^]^ Copyright 2023, American Chemical Society.

Li developed a Hofmeister gel iontronic sensor based on a chemical‐mechanical interface transduction mechanism (**Figure** **6**).^[^ ^34^ ^]^ The modulation of the mechanical properties of ATMP‐PVA hydrogels by Hofmeister effects was systematically studied to establish a quantitative analysis of the gel elastic modulus for chemical co‐solvents. The mechanical properties of hydrogels can be extensively and reversibly changed by ionically regulating the aggregation state of polymer chains, and the information of different chemical compositions can be stored in ATMP‐PVA gels. Chemo‐mechanical interfacial transduction regulated by the Hofmeister effect can respond and convert biological/chemical signals in real‐time, facilitating the gel iontronic sensor to distinguish, classify, and quantify various cations, anions, amino acids, and sugars.

### 2.2 Hofmeister Effects in Hydration Chemistry of Hydrogels

In recent years, solar evaporation technology has shown great potential in alleviating global water shortages due to its high energy conversion efficiency. Reducing the evaporation enthalpy of water is the key to improving the performance of solar desalination. Hofmeister effects have unique advantages in evaporative seawater desalination due to the tunable hydration chemistry through regulating ion behaviors. Qiu prepared a highly hydratable hydrogel network using modified needle coke as a photothermal material and PVA as a hydration matrix.^[^ ^35^ ^]^ The superior evaporative performance of hydrogels in saline stems from their hydration regulated by chaotropic Cl^−^, where Cl^−^ can regulate the hydration chemistry of PVA in hydrogels and suppress the associated crystallinity, thereby increasing the intermediate water content and decreasing the evaporation enthalpy of saline. Further mechanistic studies showed that salt ions could affect the hydrogen bond between the hydrogel skeleton and water molecules, confirming that Cl^−^ in saline could promote water activation. Saline with relatively low concentration promotes the hydrophobic interactions and density of PVA chains, making hydrogels swollen in it exhibit significantly higher high‐pressure stress than those swollen in pure water. This work demonstrates the great potential of utilizing Hofmeister effects in seawater desalination.

### 2.3 Hofmeister Effects in Temperature‐Responsive Hydrogels

Due to the significant phase transition effect of gels in response to temperature, it is of great significance to study the influence of Hofmeister effects on temperature‐responsive hydrogels.^[^ ^36^ ^]^

Lim investigated the impact of Hofmeister effects on thermogels.^[^ ^37^ ^]^ Anions and their concentrations were found to significantly affect thermal gelation, significantly lowering the sol‐to‐gel transition temperature. Kosmotropic anions can increase the hardness and viscosity of the gel phase and affect the micellization behavior of polymers by increasing the surface tension and reducing solubility, while chaotropic anions will cause the opposite effect. In addition, cations have very little effect on thermogels. Thus, regulating the type and concentration of anions may also provide another avenue for tuning the physical properties of thermogels without changing the polymer structure or concentration.

### 2.4 Hofmeister Effects on Redox Hydrogel Electrodes

Tsujimura first studied Hofmeister effects on redox hydrogel electrodes.^[^ ^38^ ^]^ Ions affect the electrochemical process of the electrode by specifically interfering with the electron transfer between enzymes and electrical signaling molecules and changing the structure of the hydrogel. The relative decrease in oxidation current is minimal in the middle of Hofmeister series and increases monotonically on both sides. The increase in ionic strength inhibits the electron transfer process of the enzyme/Os complex. In addition, the higher the polarity of the ion, the greater the dispersion of its adsorption on the uncharged part of the polymer/enzyme, and the resulting swelling of the hydrogel reduces the catalytic current. This research has implications for the design of hydrogel electrodes and the choice of electrolyte ions for bioelectronic applications.

### 2.5 Hofmeister Effects in Aerogels

Eychmüller applied Hofmeister effects to easily and rapidly prepare noble metal/alloy aerogels with controllable composition (Au, Ag, Pd, and Pt), size (3.1 to 142.0 nm), and unique morphology.^[^ ^39^ ^]^ Taking NH_4_F as the initiator to induce the gelation of gold nanoparticles as an example, the gelation time is only a few hours, and the concentration of the precursor metal salt can be as low as 0.02 mm. The sol‐gel process at the microscopic level includes: 1) the aggregation of nanoparticles under the salting‐out effect, 2) the removal of negatively charged ligands by salt cations, 3) the anisotropic fusion of nanoparticles under the combined effect of electrostatic repulsion and van der Waals force attraction, which is reflected in the growth and precipitation of aggregated nanoparticles, and 4) the formation of gel networks. Hofmeister effects are reflected both in the salting out of nanoparticles and the removal of ligands.

## 3 Hofmeister Effects in Battery Design

Finding suitable low‐cost salt solutes as high‐concentration electrolytes is crucial for developing high‐performance aqueous batteries. ClO_4_^−^, as representative chaotropes, strongly tend to break the water structure. Therefore, in Hofmeister anions, considering solubility, cost, and solvation strength, ClO_4_^−^ is a suitable electrolyte for developing low‐cost, high‐concentration, high‐voltage aqueous batteries.^[^ ^40^ ^]^

Hydrogel electrolytes are widely used in battery design. The mechanical properties and ionic conductivity of conventional hydrogel electrolytes decrease significantly after freezing, resulting in poor cycle stability and even short circuits. Wu improved the antifreezing capability of the hydrogel electrolyte by utilizing Hofmeister effects of low‐concentration Zn(ClO_4_)_2_, and realized the ultra‐low temperature Zn‐ion battery at −30 °C.^[^ ^41^ ^]^ ClO_4_^−^ can form weak hydrogen bonds with hydrogels and water molecules, thereby improving the hydrophilicity of hydrogels and making hydrogels have good mechanical flexibility at low temperatures. In addition, ClO_4_^−^ can regulate the free water content and effectively suppress side reactions and dendrite formation.

**Figure 7:**
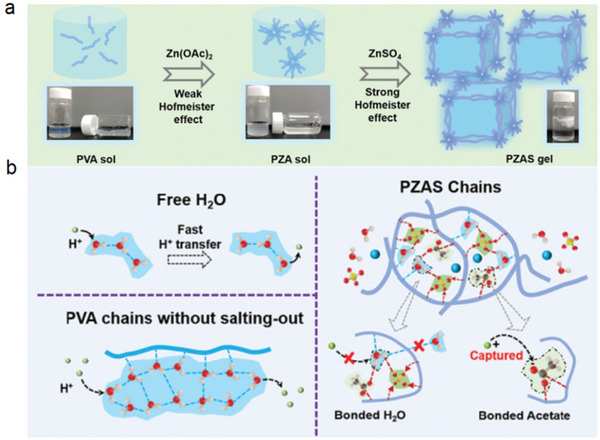
a) Schematic of PVA sol/gel after weak Hofmeister effects (by acetate) and strong Hofmeister effects (by sulfate) and digital photographs of the corresponding products. b) Mechanisms of proton transfer in free water and PVA without salting‐out; and possible ways that hinder the transfer of H^+^ in PZAS with strong salting‐out effects. Reproduced with permission.^[^ ^42^ ^]^ Copyright 2022, Wiley‐VCH.

In an acidic MnO_2_‐Zn battery, metal Zn easily reacts with the acidic electrolyte to generate hydrogen gas, which makes the acidity of the electrolyte decrease continuously, resulting in a decrease in the activity of the positive electrode and, ultimately, a decrease in battery performance. This is one of the main factors restricting the practical application of aqueous acid MnO_2_‐Zn batteries. Chen successfully developed an ultra‐low‐cost proton‐barrier separator through the powerful Hofmeister effects and significantly improved the electrochemical performance of MnO_2_‐Zn batteries (**Figure** **7**).^[^ ^42^ ^]^ The remarkable ability of this proton‐barrier separator to promote the transfer of Zn^2+^ but hinder the transfer of H^+^ comes from the salting‐out process of concentrated SO_4_^2−^, which makes the hydrogen bond network disordered and discontinuous and even forms an isolated hydrophilic cage. Its far superior performance to commercial anion‐exchange membranes can be attributed to this excellent ionic conductivity, stability, and ability to hinder H^+^ transfer. Therefore, the new functions of proton shielding and selected metal ion transport that Hofmeister effects can bring to the separator provide a new effective strategy for developing high‐performance, stable batteries.

Hu developed a hydrogel electrolyte composed of NH_4_Cl and ZnCl_2_ mixed aqueous electrolyte.^[^ ^43^ ^]^ The interfacial adhesion strength of the near‐neutral hydrogel electrolyte is significantly enhanced, due to the binding and hydrophobic interactions of chitosan chains generated by Hofmeister effects induced by NH_4_^+^ and Cl^−^, and has facilitated interfacial charge transfer dynamics. This work provides a simple and effective strategy to integrate high interfacial adhesion strength, high ionic conductivity, and low corrosion into one hydrogel electrolyte by exploiting Hofmeister effects of the electrolyte, thereby enhancing the electrochemical performance and mechanical durability of batteries.

## 4 Hofmeister Effects in Nanosynthesis

### 4.1 Hofmeister Effects in the Synthesis of Metal Nanoparticles

Pileni used copper nanocrystals as a template to study Hofmeister effects of anions on the shape control of the prepared nanocrystals.^[^ ^44^, ^45^ ^]^ The results show that other types of Hofmeister anions are not selectively adsorbed on the specific crystal planes except for F^−^ and Cl^−^. The control of F^−^ and Cl^−^ on the shape and growth of nanocrystals is closely related to the adsorption crystal plane, adsorption strength, and ion concentration, while Hofmeister effect does not play a role in the growth of nanocrystals.

Grzybowski further studied the effect of salts on the aggregation effect of gold nanoparticles and found that not only Hofmeister anions had no impact on the precipitation tendency, but also the cation‐induced precipitation did not conform to Hofmeister series and was independent of the size of the hydrated cations.^[^ ^46^ ^]^ Thanh studied the fine‐tuning effect and plasmonic properties of Hofmeister anions on the growth aspect ratio of gold nanorods.^[^ ^47^ ^]^ This effect depends both on the interaction between nanoparticles and ions, and on the interaction between ions and surfactants. Anions with a high affinity for gold, such as Br^−^, produce nanorods with smaller aspect ratios, while anions with a lower affinity for gold, such as NO_3_^−^, HSO_4_^−^, Cl^−^ will produce nanorods with higher aspect ratios.

### 4.2 Hofmeister Effects in the Synthesis of Porous Materials

Yu et al. studied the regulating effect of anions on the crystallization process of zeolite.^[^ ^48^ ^]^ SO_4_^2−^ and F^−^ can well activate water molecules in the cation hydration shell, while Cl^−^, Br^−^, I^−^ and SCN^−^ can stabilize the water molecules in the cation hydration shell following Hofmeister series. Xia demonstrated that a series of Hofmeister anions regulate the formation of ZIF‐8 by affecting ammonia concentration. Such regulation is based on the various interaction with Zn^2+^ and H^+^ of Hofmeister anions with different sizes and dehydration effects.^[^ ^49^ ^]^

Gu developed a series of strategies for MOFs synthetic methodology based on Hofmeister effects. They first revealed that salt‐in species are highly favorable for the solubility of organic ligands, thus inducing the self‐assembly of MOFs.^[^ ^50^ ^]^ They constructed ordered mesoMOFs and demonstrated a salt‐mediated templating strategy by coupling Hofmeister effects with the structure‐directed properties of triblock copolymers.^[^ ^51^, ^52^ ^]^ The synergistic effect based on the triblock copolymer template and Hofmeister anions promotes the nucleation of the stable MOF in the aqueous phase and the in situ crystallization of the MOF around the template, resulting in large mesoporous crystallites with periodic arrangements. More recently, they have demonstrated that nanoemulsions composed of double surfactants and hydrophobic aromatic compounds exhibit excellent versatility as soft templates with the help of Hofmeister anions, and have prepared layered super‐large mesoporous MOF with flexible architectures.^[^ ^53^ ^]^ Nanoemulsion and Hofmeister ions have a synergistic effect. After removing ClO_4_^−^, only particles with sparse pits were obtained, indicating that the interaction between the surfactant and the MOF precursor was too weak to support the emulsion‐directed effect. Furthermore, Murakami reported that the growth size trend of COF crystals follows Hofmeister effects of ionic liquids and Guttman donor numbers.^[^ ^54^ ^]^

### 4.3 Hofmeister Effects in the Synthesis of Polymersomes

**Figure 8:**
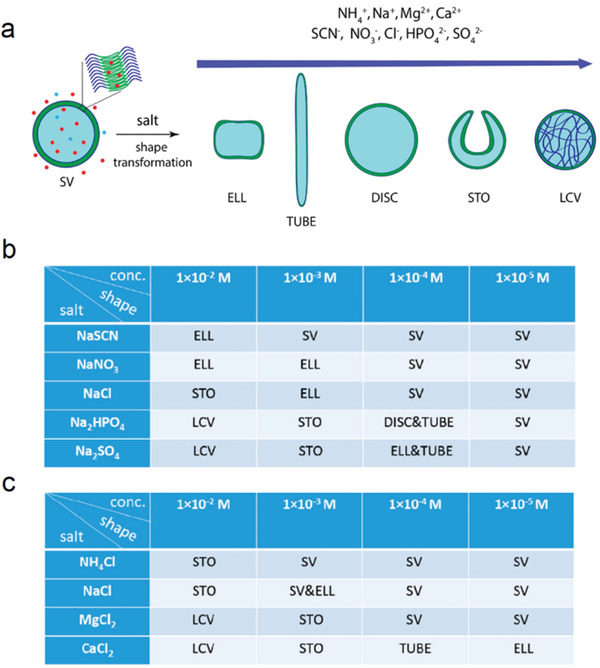
a) After addition of salts containing various cations (NH_4_^+^, Na^+^, Mg^2+^, Ca^2+^) and anions (SCN^−^, NO_3_^−^, Cl^−^, HPO_4_^2−^, SO_4_^2−^) into the polymersome solution, the SV shape changed to ellipsoid (ELL), tube, disc, stomatocytes (STO), and large compound vesicles (LCV). The capacity of ions to induce these shape change follows the order of Hofmeister series, as the arrow pointed. b) Shapes obtained from various sodium salts at different concentrations (10^−2 _^ 10^−5^ m). The TEM images of SV, ELL, STO, DISC, and LCV (cryo‐TEM image inserted); scale bar (red): 500 nm. c) Shapes obtained from variation of the cations at different the concentration ranging from 10^−2^ to 10^−5^ m. Reproduced with permission.^[^ ^55^ ^]^ Copyright 2020, American Chemical Society.

The size and shape control of polymersomes are critical for their applications in nanomedicine and micro/nanorobots. Wilson explored the use of Hofmeister effects to control the shape of polymersomes (**Figure** **8**).^[^ ^55^ ^]^ By adjusting the type and concentration of ions, vesicles of different shapes were synthesized, induced not only by osmotic pressure but also by the interaction of ions with the polymersome membrane. Interestingly, the ability of anions to induce polymersome shape changes follows Hofmeister series, whereas the ability of cations to induce polymersome shape changes is opposite to Hofmeister series. Kosmotropic ions are more powerful at reshaping polymersomes than chaotropic ions because they are more polarizable and hydrated. Therefore, the shape can be changed efficiently at very low concentrations, which is suitable for encapsulating ion‐sensitive particles. Chaotropic ions can generate osmotic pressure at relatively high concentrations without causing significant shape changes, making them suitable for crystallization applications.

## 5 Hofmeister Effects in Nanomotors

Chemotaxis is the directional movement behavior of motile cells or particles stimulated by chemical gradients.^[^ ^56^, ^57^ ^]^ The extent and direction of chemotactic movement depend on very specific interactions between nanomotors and solutes.

**Figure 9:**
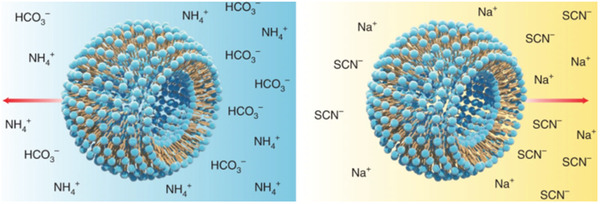
Hofmeister effects‐driven chemotaxis of nanomotors Reproduced with permission.^[^ ^59^ ^]^ Copyright 2019, Springer Nature.

Sen, Velegol, and Cremer studied the chemotaxis caused by Hofmeister effects between liposomes and solutes (**Figure** **9**).^[^ ^58^ ^]^ POPE liposomes at a molar ratio of 70:30 in an (NH_4_)_2_CO_3_ gradient exhibited negative chemotaxis. Silica particles coated with specific phospholipids also exhibit a similar motion to liposomes, while uncoated particles do not exhibit chemotactic motion. This Hofmeister effects‐driven liposome chemotaxis depends on the chemical composition of the liposome and the presence of solute ions. Developing biomacromolecule‐based nanomotors with stronger Hofmeister effects‐driven chemotaxis that can move efficiently toward the electrolytic physiological environment is a vital task for future biomedical applications of nanomotors.

## 6 Applying Hofmeister Effects to Develop Ion Sensors

### 6.1 Electrochemical Ion Sensors

Conventional ion sensors based on ion‐selective electrodes achieve high sensitivity by using ion‐selective layers or recognition receptors that specifically interact with ions to be measured.^[^ ^60^, ^61^, ^62^, ^63^, ^64^, ^65^, ^66^ ^]^

In general, electrochemical ion sensors are based on electrostatic interaction, chaotropic/kosmotropic effect at the electrode interface, ion/molecular recognition strategy, etc., to enhance the sensitivity, selectivity, specificity, and stability of ion sensing.^[^ ^67^, ^68^, ^69^, ^70^, ^71^, ^72^, ^73^, ^74^, ^75^, ^76^ ^]^

Hofmeister effects in the design of ion‐selective electrodes are very important not only for fundamental electroanalytichemistry but also of great significance in ion sensor engineering. Recently, Lisensky presented a laboratory case to illustrate this.^[^ ^77^ ^]^ Students assessed ion selectivity by assembling, calibrating ion‐selective electrodes, testing electrode performance in interference solutions, and comparing with Hofmeister series. Hofmeister effects in the most straightforward electrochemical process were explored by selecting a Hofmeister anion‐selective electrode and various interfering ions for testing. The measured selectivity and response curves will help students better understand Hofmeister effects in electrochemical ion sensors.

### 6.2 Ion‐Sensitive Field‐Effect Transistors

Ion‐sensitive field‐effect transistors (ISFET) are microelectronic ion‐selective devices with dual characteristics of electrochemistry and transistor. Compared with traditional ion‐selective electrodes, it has the following advantages: 1) High sensitivity, fast response, and convenient detection. The input impedance is high, the output impedance is low, and it has the function of impedance transformation and signal amplification, which can avoid the interference of external induction and secondary circuit; 2) Small in size and light in weight, especially suitable for dynamic monitoring in living organisms; 3) Not only can the miniaturization of a single device be realized, but also the integration of various ions and multifunctional devices can be realized by using integrated circuit technology and micromachining technology, which is suitable for mass production, low cost, and has the advantages of miniaturization and integration development potential; 4) It can realize all‐solid structure, high mechanical strength, wide application range and strong adaptability; 5) It is easy to match with the external circuit, easy to use, and can be connected with the computer to realize online control and real‐time monitoring; 6) The sensitive materials are extensive. Especially its miniaturization characteristics make it of great significance in the field of biomedical engineering, which can quickly and accurately detect the concentration of trace ions in the physiological environment.

Kimura first reported the ISFET anion‐sensing membrane that can selectively recognize Cl^−^.^[^ ^78^ ^]^ Doping of polyions complexed with the corresponding quaternary ammonium chlorides and appropriate annealing temperature increase the selectivity of Cl^−^ over other Hofmeister anions.

Clément has developed a nanoscale ISFET that can measure changes in cation concentrations in various aqueous solutions and achieve highly sensitive measurements of cation concentrations in blood serum.^[^ ^79^ ^]^ Traditional ISFET often requires an ion‐selective layer (such as a pH sensor) for each ion to be tested to identify and detect specific ions, resulting in structural and system complexity. In this work, multiple ions can be measured without using an ion‐selective layer by using ionic responses unique to nanoscale surfaces. Although the mechanism has not been elucidated, it should be related to the chemically mediated non‐coulomb interaction of ions on the surface. This work is of great value for developing layer‐free ion detection devices in the future.

### 6.3 Hydrogel Ion Sensors

Li proposed the elastic sensing of hydrogels based on Hofmeister effects and the electrical sensing based on the migration of hydrated ions.^[^ ^80^ ^]^ Kosmotropic agents lead to an increase in the mechanical strength of the hydrogel, while chaotropic agents have a weakening effect. The conductivity of hydrogels can be tuned by co‐solvent loading and tensile stress‐strain manipulation. This provides a good idea and model for developing sweat detection sensors with excellent performance.

### 6.4 Colorimetric Ion Sensors

By modifying gold nanoparticles with ionic liquids, Liu developed an As^III^‐specific probe with significantly improved sensitivity due to Hofmeister effects.^[^ ^81^ ^]^ Only in SCN^−^ and NO_3_^−^ buffers did Au NPs show a response to As^III^, possibly due to the strong Hofmeister effects of the ions, leading to enhanced particle aggregation. However, the Hoffmeister effect of SCN^−^ is powerful, leading to a significant blank response of the probe, so NO_3_^−^ buffer is the best choice.

### 6.5 Nanopore Ion Sensors

This part has been discussed in very detail in our previous work.^[^ ^19^ ^]^

## 7 Hofmeister Effects in Supramolecular Chemistry

Hofmeister effects are widely used in supramolecular chemistry, especially in the fields of supramolecular self‐assembly and anion recognition/transport.^[^ ^82^, ^83^, ^84^, ^85^, ^86^, ^87^, ^88^ ^]^

Ulijn first systematically demonstrated Hofmeister effects in enzyme‐instructed supramolecular self‐assembly and hydrogelation of peptides.^[^ ^89^, ^90^, ^91^ ^]^ Enzyme catalysis acts as a kinetic factor, and specific ion interactions and protein templates act as thermodynamic contributions during hydrogelation. Hydrophobic interactions lead to chiral differential organization and supramolecular structure formation. Therefore, specific salts can regulate the complex self‐assembly behavior of enzyme‐instructed hydrogels. Salts affect enzyme kinetics and the corresponding nucleation and growth of nanostructures by changing the enzyme's structure. This is manifested in different hydrophobic interactions, different orders and chiralities of the resulting gel‐phase materials, and variable mechanical properties. The order of efficiency with which ions facilitate this interaction follows Hofmeister trend, and this specific ion effect can be attributed to the synergistic outcome of electrostatic and hydrophobic interactions. Such a strategy has been widely used in discovering supramolecular self‐assembled peptide hydrogels with various characteristics like mechanical properties.^[^ ^92^ ^]^

Schatz reported using atomic molecular dynamics simulations to explore Hofmeister effects to control the structure of self‐assembled peptide nanofibers.^[^ ^93^ ^]^ The results show that the formation of *β*‐sheets in nanofibers follows Hofmeister effects. This is due to the closer interaction of ions and residues with similar chaotropic nature leading to the formation of stable *β*‐sheets inside the peptide near the core of the nanofibers. In addition, strongly hydrated ions can induce coil‐to‐*β*‐sheet transition by forming salt bridges between lysine residues, thereby improving the mechanical stability of nanofibers. Intermolecular interactions between anions and residues affect how these ions are distributed, affecting nanofiber size, morphology, and conformation. The interaction of weakly hydrated ions (I^−^ and Br^−^) with nonpolar amino acid residues allows the presence of these large anions between most of the *β*‐sheet residues in the nanofibers, thereby breaking hydrogen bonds and making these structures less stable. On the other hand, strongly hydrated ions (F^−^ and Cl^−^) are more easily attracted to the positively charged lysine residues on the nanofiber surface, leading to chain aggregation to form hydrogen bonds and further formation of *β*‐sheets.

In addition, Li et al. used the short peptide nanofibers formed after the phase transition to mediate the anisotropic water transport in the gel and found that the effect of anions on the water transport rate followed Hofmeister effects.^[^ ^94^ ^]^ The size limitation of the channels in the nanofibers prevents the nanoparticles from being transported with water or ions so that the ions in an aqueous solution are separated from the nanoparticles. This system exploits the function of supramolecular hydrogel materials to separate trace nanoparticles.

**Figure 10:**
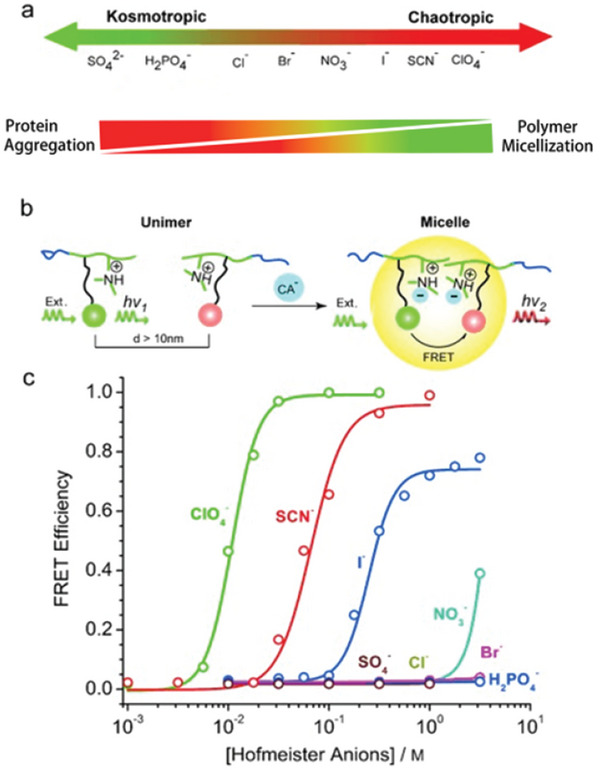
a) Chaotropic anions induce micelle self‐assembly of PEO‐b‐PR copolymers with protonated PR segment, which is a reversed effect (salt‐out) with respect to their ability to solubilize proteins (salt‐in). b) Illustration of FRET design to investigate CA‐induced micelle self‐assembly. Addition of CA results in micelle formation and efficient energy transfer from donor (TMR) to acceptor (Cy5) dyes. c) Chaotropic anion‐induced micelle self‐assembly showing the anti‐Hofmeister trend. Reproduced with permission.^[^ ^95^ ^]^ Copyright 2014, Wiley‐VCH.

In addition to self‐assembled supramolecular multimolecular materials, Gao also studied Hofmeister effects‐induced supramolecular self‐assembly of ionic polymeric micelles (**Figure** **10**).^[^ ^95^ ^]^ Unlike chaotropic anions such as ClO_4_^−^, kosmotropic anions such as SO_4_^2−^ do not induce micelle formation. The self‐assembly process is achieved by block copolymers of chaotropic anions and ammonium groups containing hydrophobic cations. The resulting micelles containing many ion pairs provide an excellent model system to study supramolecular self‐assembly through the interaction of non‐covalent forces such as electrostatic, van der Waals, and hydrophobic interactions in aqueous environments. This also opens up the future for developing stable ionic micellar systems to deliver charged drug molecules.

Nau first proposed that dodecaborate anions of type B_12_X_12_^2−^ and B_12_X_11_Y^2−^ could form strong *γ*‐cyclodextrin complexes driven by chaotropic effects.^[^ ^96^ ^]^ According to this effect, chaotropic anions have an intrinsic affinity for hydrophobic cavities in an aqueous solution. The salting‐out effect shows that dodecaborate is a superchaotropic divalent anion. Its solubilizing ability even exceeds the two chaotropic anions, SCN^−^ and PF_6_^−^, so dodecaborate can be classified as a “superchaotropic” anion. On this basis, Nau et al. reported that spherical dodecaborate clusters could be used as anionic species carriers to load a variety of hydrophilic molecules for transmembrane transport of hydrophilic molecules.^[^ ^97^ ^]^ It solves the difficulties faced by the membrane transport of hydrophilic substances as therapeutic compounds or labeled probe molecules and the easy aggregation of charged amphiphilic carriers leading to non‐specific membrane dissolution. This borate cluster can load and penetrate cationic/electrically neutral polypeptides, amino acids, neurotransmitters, vitamins, antibiotics, and drugs through lipid membranes. Mechanical transport studies have found that transport capacity comes from the superchaotropic effect of boron cluster anions. In addition, clusters can improve the cytoplasmic uptake ability of various biomolecules. This widely adaptable superchaotropic drug molecule carrier delivery system opens the door for studying cell biology, neurobiology, physiology, and pharmacy.

Similarly, Bauduin controlled the self‐assembly process of PNIPAM chains by utilizing the adsorption of anionic polyoxometalates on uncharged PNIPAM chains induced by the superchaotropic effect.^[^ ^98^ ^]^ Therefore, strategies to produce functional nanomaterials based on the superchaotropic ions may be general.

## 8 Hofmeister Effects in Colloid and Interface Science

The impact of Hofmeister effects on colloid and interface science is enormous, especially its interaction with interface properties and its impact on colloidal stability. The colloidal stability of nanosystems is affected by two aspects of Hofmeister effects: charge and chaotropicity.^[^ ^99^, ^100^, ^101^ ^]^

Under certain circumstances, changes in these two factors will reverse Hofmeister effects.^[^ ^102^ ^]^ In addition, counterions are one of the major reasons for the restabilization phenomenon, provided that their local concentration is sufficiently high, which is related to the degree of hydrophilicity of the particle surface, the degree of hydration of the ion, and the entropy effect of the ion around the surface.

Barcikowski explored the stabilizing effect of Hofmeister effects on gold nanoparticles, which cannot be explained well by the classical DLVO theory.^[^ ^103^ ^]^ The specific ionic effect of ligand‐free aqueous gold nanoparticles is critical in biomedical or catalytic applications, as gold colloidal particles are significantly affected by the species rather than the number of ions. The stability of ligand‐free gold nanoparticles is inversely proportional to the hydration of Hofmeister anions. Adsorption of chaotropic anions (Br^−^, SCN^−^ or I^−^) at the interface leads to repulsive interactions between the gold particles. On the other hand, lyophilic anions (F^−^ or SO_4_^2−^) appear to destabilize gold colloids, while Cl^−^ and NO_3_^−^ induce moderate stabilization.^[^ ^104^ ^]^ Furthermore, they noticed that specific anion effects and pH effects influence the size and yield of Au nanoparticles <3 nm synergistically and significantly during pulsed laser fragmentation in liquids route.

Du used Surface‐Enhanced Raman Scattering to realize the ultrasensitive detection of various anions in water on the substrate of positively charged silver nanoparticles, and its sensitivity followed Hofmeister series.^[^ ^105^ ^]^ Schreiber studied the effect of adding salt on the stability of a colloidal gold nanoparticle‐protein system.^[^ ^106^ ^]^ The results showed that adding various salts did not change the respective stability of proteins or colloids in the solution. However, in binary mixtures, the stability of colloid‐protein mixtures is significantly dependent on the nature of the salts: chaotropic salts (NaSCN, NaClO_4_, NaNO_3_, MgCl_2_) stabilize the system with increasing salt concentration, while kosmotropic salts (NaCl, Na_2_SO_4_, NH_4_Cl) cause colloids to aggregate with increasing salt concentration. These observations suggest that the Hoffmeister effect can be enhanced in two‐component systems, that is, the modification of ions on the colloidal interface significantly alters the efficient protein‐mediated depletion interaction.

Bunkin investigated the ion‐specific mechanism of nanobubble stabilization in various saline solutions.^[^ ^107^ ^]^ It has been shown that the stabilization of nanobubbles is achieved by the adsorption of chaotropic anions at the interface, while the influence of cations is weaker. An increase in temperature destabilizes the adsorption state of ions, causing the nanobubbles to destabilize and disappear.

Szilagyi studied the ion‐specific effects on the colloidal stability of titania nanosheets.^[^ ^108^ ^]^ The adsorption of anions affects the resistance of nanoparticles to salt‐induced aggregation by significantly changing the surface charge characteristics. The destabilization of particles by monovalent anions can be ordered according to Hofmeister series in acidic solutions. However, the behavior of H_2_PO_4_^−^ and HPO_4_^2−^ is atypical in that their post‐adsorption coordination leads to the accumulation of negative charges on the particles resulting in charge inversion. Multivalent anions destabilize oppositely charged particles more efficiently, and the aggregation process follows the Schulze‐Hardy rule. On the other hand, when anions are adsorbed, if the surface charge density is high enough (such as Fe(CN)_6_^3−^ and Fe(CN)_6_^3−^), it will lead to particle restabilization. These results indicate that non‐DLVO forces are found only when anions (H_2_PO_4_^−^, HPO_4_^2−^, Fe(CN)_6_^3−^ and Fe(CN)_6_^4−^) interact strongly with the nanosheets.

Choi investigated the effect of Hofmeister ions on the film‐forming process of metal‐phenolic species.^[^ ^109^ ^]^ It was found that cations determine the kinetics of film growth but are not correlated with Hofmeister series. Anions are the decisive factors affecting film growth kinetics and film properties, which are related to the reverse Hofmeister series of anions. In the presence of chaotropic anions such as Br^−^, thick, rigid, but porous films are formed. On the other hand, in the presence of kosmotropic anions such as SO_4_^2−^, the growth of the film is restricted and forms a thin, soft but dense film. Thayumanavan elucidated Hofmeister effects on the size and guest encapsulation stability of polymer nanogels and explored the changes in the size and host‐guest behavior of polymer aggregates in the presence of salt.^[^ ^110^ ^]^ Anions still play a decisive role in this process, while cations do not matter. Kosmotropic anions mainly affect the size of the aggregates, while chaotropic anions cause inter‐aggregate cross‐links, leading to higher guest encapsulation stability in the nanogels. Using Hofmeister effects to fine‐tune the size and encapsulation stability of polymer nanogels has important guiding significance for synthesizing polymer nanomaterials. It also promotes their applications in drug delivery and sensing.

Cicuta explored the effect of Hofmeister monovalent cations on the gelation kinetics of silica nanoparticles, with trends consistent with Hofmeister series.^[^ ^111^ ^]^ From Cs^+^ to Li^+^, there is an order of magnitude difference in the gelation time. Non‐DLVO hydration forces dominate particle‐particle interactions at the level of hydration ion diameters, paving the way for the regulation of nanoparticle gelation using Hofmeister effects. Zhu examined the effect of adding different types of ions on the interaction of semihydrophobic nanoparticles with a supported phospholipid bilayer in aqueous media.^[^ ^112^ ^]^ By adding different anions at the same ion concentration to the interface, it was observed that the growth rate of the nanoparticle‐induced lipid‐poor regions followed Hofmeister series, suggesting that anions regulate hydrophobic interactions. In contrast, the results for specific cations do not follow Hofmeister series but show a trend of Cs^+^ ∼ Rb^+^ > Na^+^ ≫ N(CH_3_)_4_^+^. Colloid‐lipid interactions can be tuned by the addition of specific ions to control the rate of morphological change of the lipid bilayer, thereby enhancing the delivery of nanomaterials across biomembranes and weakening unwanted interfacial interactions, by tuning ions in the aqueous phase environment to minimize nanomaterial cytotoxicity.

## 9 Hofmeister Effects in Nanomedicine

### 9.1 Hofmeister Effects in Drug Delivery

Flanagan first studied the effects of various anions on the water solubility of model drugs and the encapsulation efficiency of microspheres.^[^ ^113^ ^]^ Hofmeister anions reduced the solubility of drugs, with kosmotropes such as ClO_4_^−^ and SCN^−^ having the greatest reduction and the least effect on DCM solubility, resulting in microspheres with high drug loading. Although SO_4_^2−^ would reduce the solubility of the drug, it would also reduce the water solubility of DCM, thus not increasing the drug loading of the microspheres. Khashab reported Hofmeister effects of inorganic salts on the loading of doxorubicin on nanodiamonds of different sizes.^[^ ^114^ ^]^ NaCl, NaNO_3_, Na_2_SO_4_, KCl, CaCl_2_, (NH_4_)_2_SO_4_ were found to increase the drug loading on small‐size nanodiamonds (5–10 nm).

**Figure 11:**
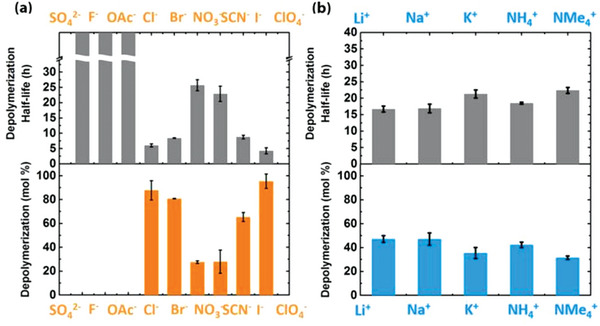
Summary of anion (1 m) and cation (0.02 m) specific effect on depolymerization kinetics for microcapsules suspended in methanol at [TFA] = 0.01 m as represented by depolymerization half‐life tD50 and depolymerization mol % at 16 h. a) Anion specificity in the coactivation (cation = lithium), showing that only chaotropic anions accelerated the depolymerization rates. The tD50 SO_4_^2–^, F^–^, and OAc^–^ were marked in break columns because these values exceeded the measuring scale and no depolymerization was observed over 48 h. b) Cation specificity in the coactivation (anion = chloride), showing a modulating effect on the depolymerization rates. Reproduced with permission.^[^ ^115^ ^]^ Copyright 2017, American Chemical Society.

Specific ion‐activated stimuli‐responsive materials are highly important for designing chemical logic gates and signal amplification schemes in biomedical applications. Identifying specific ion effects as triggers for releasing encapsulated drug microcapsules is a highly advantageous and promising strategy. Moore systematically investigated the specific ion coactivation effect at the solid‐liquid interface of transient polymer microcapsules and developed cPPA programmable microcapsules whose load‐based release rate can be tuned by Hofmeister effects (**Figure** **11**).^[^ ^115^ ^]^ Hofmeister effects can accelerate the depolymerization of the cPPA interface in weakly acidic solutions, and these ions did not independently trigger the depolymerization of cPPA. However, they exhibited a coactivation behavior with the acid. The depolymerization rate can be tuned according to the ion species, which is related to Hofmeister effects. Anions are the dominant factor, and cations mediate the coactivation effect by pairing with anions, where weaker ion pairs lead to stronger coactivation. Using Li^+^ as the countercation to change the anion species, no co‐activation effect was observed for the kosmotropic anions. However, in situ ion‐exchange experiments found that the coactivation behavior changes abruptly at the boundary between the kosmotropic anions and the chaotropic anions, which is consistent with Hofmeister effects. Therefore, the interaction between the chaotropic anions and cPPA is more favorable for coactivated depolymerization. The dominance of anions over cations may be attributed to their stronger interaction with the interface. As the solvation degree of anions decreases from Cl^−^ to ClO_4_^−^, the coactivation effect decreases from Cl^−^ to NO_3_^−^ and increases from SCN^−^ to ClO_4_^−^, showing a non‐monotonic trend. Two main mechanisms may be responsible for this effect: ionic effects stabilizing the depolymerization intermediates (Cl^−^ and Br^−^) and electrostatic effects polarizing the shell‐wall interface (SCN^−^, I^−^, ClO_4_^−^). Furthermore, unlike LiCl, LiSCN exhibits a saturation‐type concentration effect attributed to the electrostatic screening effect induced by the highly charged surface of the weakly solvated SCN^−^. This work is of great significance and guiding value for designing stimuli‐responsive drug release platforms based on Hofmeister effects in the future.

### 9.2 Hofmeister Effects in Molecular Imaging

**Figure 12:**
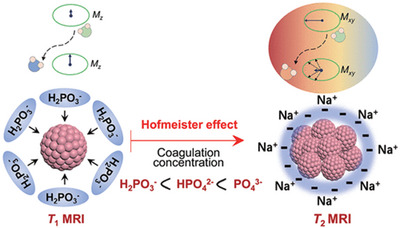
Hofmeister Effects‐Based T_1_–T_2_ Dual‐Mode MRI. Reproduced with permission.^[^ ^116^ ^]^ Copyright 2019, American Chemical Society.

Using Hofmeister effects to design stimuli‐responsive materials for physiological environments is a very useful and important strategy. Zhao designed functional Fe_2_O_3_ nanoparticles responsive to Hofmeister effects in the tumor microenvironment as a contrast agent for T_1_‐T_2_ dual‐modal MRI (**Figure** **12**).^[^ ^116^ ^]^ When the self‐assembled Fe_2_O_3_ nanoparticles were dispersed and degraded by an acidic environment and GSH, the longitudinal relaxation efficiency r_1_ was greatly improved. Then, the phosphate‐induced Hofmeister effect causes a significant increase in the transverse relaxation efficiency r_2_. Studies have shown that Fe_2_O_3_ nanoparticles will aggregate with prolonged reaction time in the tumor microenvironment. This aggregation is closely related to Hofmeister effect of phosphate and its hydrogen bond network in an aqueous solution. In the first stage, due to the decomposition of Fe_2_O_3_ nanoparticles, their particle size gradually decreases. However, in the second stage, the particle size increased significantly. With the detachment of ligands on the surface of Fe_2_O_3_ nanoparticles, phosphate (mainly in the form of H_2_PO_3_^−^) can be adsorbed on the exposed Fe_2_O_3_ nanoparticles through coordination, thereby triggering the aggregation of Fe_2_O_3_ nanoparticles. Since the magnetic dipole moment of the aggregated nanoparticles has a higher saturation magnetic moment and larger particle size than the initially assembled Fe_2_O_3_, it facilitates the enhancement of T_2_ contrast.

## 10 Hofmeister Effects‐Mediated Transport Behaviors in Nanosystems

How the transport of charged nanoparticles in transport nanopores is affected by oppositely charged ions remains poorly understood. Chen explored the effect of cations on the transport of graphene oxide‐based nanomaterials in porous media.^[^ ^117^ ^]^ The results show that the inhibitory effect of ions on transport follows Hofmeister series, and ions with larger radius have stronger interactions. In addition, the electrostatic interaction and coordination between cations and the material surface are also crucial to transport. On this basis, the influence of Hofmeister anions on the transport laws of positively charged nanomaterials needs to be further explored.

**Figure 13:**
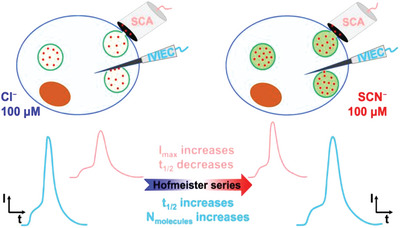
Chemical Storage and Exocytotic Dynamics Detected Amperometrically Regulated by Hofmeister Anions. Reproduced with permission.^[^ ^118^ ^]^ Copyright 2022, American Chemical Society.

Using amperometry to study the kinetics of anion transport across cell membranes is of great significance for the understanding of pharmacological and pathological processes. Ewing studied the effect of anions on regulating the kinetics of chemical storage and transmembrane transport within nanovesicles using amperometry (**Figure** **13**).^[^ ^118^ ^]^ The results showed that the released fraction of vesicles in secreting cells decreased after chaotropic anion treatment. Furthermore, treated cells had opposite effects on the kinetics of vesicle release and opening due to altered protein physicochemical properties.

## 11 Outlook

Hofmeister effects has been employed as a powerful tool in nanotechnology. We believe the following comments and suggestions will be very constructive and useful for “playing Hofmeister games” in nanosystems.

1. Most nanosystems based on Hofmeister effects use water as the solvent. Though these are very important for biomedical applications, studying Hofmeister stories in non‐aqueous solutions is still of great significance.^[^ ^119^, ^120^ ^]^ Uncovering ion‐specific effects in non‐aqueous solvents is expected to guide the exploration of new colloidal systems, the engineering of new environments for the stability and functionality of nanoparticles, and the preparation of sustainable electrolytes using non‐aqueous solvents. Furthermore, these will contribute to developing a gold standard theory for describing electrostatic interactions in concentrated ion environments.
2. Hofmeister effects have shown tremendous power in improving the properties of hydrogels, especially the mechanical properties. This enables hydrogel fabricated via Hofmeister effects with huge potential to act as high‐performance flexible sensors. The reversibility and biocompatibility further provide natural advantages of sensing applications. In addition, Hofmeister effects are still one of the most critical factors affecting hydrogel engineering. Thus, it is very necessary to explore deeper the mechanisms and details of how Hofmeister effects change the key properties and parameters of hydrogels.
3. For battery design, it would be interesting if Hofmeister effects could be strategically utilized to overcome the traditional drawbacks of batteries, especially for those aqueous batteries restricted by the factors such as concentration, temperature, pH, etc. Moreover, developing hydrogel electrolytes rationally based on Hofmeister effects is beneficial for improving the electrochemical performance and mechanical properties of batteries. Hofmeister effects can help to improve the adhesion strength of the hydrogel electrolyte on the electrode surface, achieving reliable and firm mechanical adhesion and effectively reducing the interfacial charge transfer resistance. Applying Hofmeister effects to develop proton‐barrier separators with selected ion transportation is also meaningful for designing high‐performance stable batteries.
4. Using Hofmeister effects to guide nanosynthesis is also a big topic that still needs much more attention. As mentioned above, many excellent works have been reported. Since Hofmeister effects can affect and participate in various parts of nanosynthesis, it is imperative to widely explore the roles and rules of Hofmeister effects from different perspectives such as material chemistry, crystallography, colloidal and interfacial chemistry, physical chemistry, etc., based on different nanomaterials and corresponding synthetic methodology. On the one hand, Hofmeister effects can be employed to guide the nanosynthesis through selective ion absorption on the nanomaterials. On the other hand, it is quite necessary to consider all other physicochemical of ions in the process of nanosynthesis and other conditions in the synthesis such as pH, agents, solvents, etc. Exquisitely utilizing Hofmeister effects will be very helpful for functional nanosynthesis with high quality. Nevertheless, the inappropriate addition of Hofmeister ions will result in undesirable products or unfavorable reaction condition. Based on the current work, setting the concentration gradient of various Hofmeister ions in certain cases of nanosynthesis to explore the specific regulation “tricks” is strongly recommended for preliminary study.
5. Developing nanomotors/robots that can work efficiently in the physiological environment is the most important task in this field. Hofmeister effects could facilitate a lot to power nanomotors/robots via chemotaxis. Enzyme‐powered nanomotors/robots, vesicles, polymer‐based nanomotors/robots, liposomes, and artificial cells are ideal candidates for such applications. It is of great significance to develop nanomotors/robots based on Hofmeister effects in the physiological environment for in vivo applications as it will bring revolutionary breakthroughs to nanomedicine. However, designing such nanomotors/robots is extremely challenging due to the weak response and differentiation of nanomotors/robots toward Hofmeister anions, especially in the complex physiological environment.
6. As ion‐specific effects, Hofmeister effects are gifted for the ion detection applications such as ion sensors and anion recognition. Designing appropriate systems that can be responsive to certain ion species according to Hofmeister effects is the key to improving the sensitivity and selectivity. It is worthwhile mentioning here that such merit is not limited to chemistry and has a bright future in bioelectronic applications.
7. Hofmeister effects have been more and more promising in supramolecular chemistry. For supramolecular self‐assembly systems, it can significantly affect the nanostructures of self‐assembled peptides or polymers. Additionally, Hofmeister effects are highly useful for studying host‐guest chemistry which is particularly conducive to building up supramolecular host‐guest nanosystems.^[^ ^82^, ^120^, ^121^, ^122^ ^]^
8. Superchaotropic boron clusters developed by Nau that can be used as broadband membrane carriers have unlimited application potential and research value in biochemistry, biomedical engineering, and supramolecular chemistry.^[^ ^98^ ^]^ It opens a new door for drug delivery, especially for transporting hydrophilic cargos.
9. Decoding Hofmeister effects in the colloidal and interfacial systems is to clarify the tricky waters in the nanosystems and understand the stabilization mechanism of nanoparticles in electrolyte solutions. This will also provide valuable information for studying the physical chemistry of nanosystems.
10. Making ions as the stimuli to facilitate smart nanomedicine is wonderful, and Hofmeister effects contribute a perfect platform to this. Particularly, designing novel ion‐responsive nanosystems, which include controllable coagulation of inorganic nanomaterials, ion‐induced supramolecular self‐assembly and gelation, ion‐triggered depolymerization of microcapsules, ion‐activated chemotaxis, and transport process, etc. is very promising for enhanced therapeutics and diagnosis.
11. As for investigating the Hofmeister mystery in the transport process, it is very attractive to study cellular transport from the biophysical perspective and transport in porous nanomaterials from a chemical engineering perspective. Understanding how Hofmeister effects regulate cellular transmembrane transport is the key to studying many biochemical and biophysical problems. While studying how Hofmeister effects are involved in the transport of porous nanomaterials will lead to a better design of porous nanomaterials and further improve their surface functionalities and performance.
12. The presence of anti‐Hofmeister effects could not be ignored. Among the dozens of series reported in the last century, although there are a small number of counterexamples, the positions of ions in most series are consistent. The existence of reverse Hofmeister effects shows that these properties are not governed by exactly the same rules, but are determined by specific systems. Undoubtedly, the electrostatic interaction of the ions with the solution is the dominant factor. Under some special conditions, such as when the pH value in the solution is lower than the isoelectric point, the effect of a low concentration of anions on protein solubility follows anti‐Hofmeister effects. Gibb and Beer use host‐guest chemistry as a powerful tool to unveil the mystery of anti‐Hofmeister effects.^[^ ^17^, ^123^ ^]^ For Hofmeister anions, the size discrimination and anion‐induced interfacial and aggregation effects play key roles, especially in the systems with specific anion selectivity which do not follow the classical Hofmeister series.

## Conflict of Interest

The authors declare no conflict of interest.
